# Cost-effectiveness of Atezolizumab Plus Bevacizumab vs Sorafenib as First-Line Treatment of Unresectable Hepatocellular Carcinoma

**DOI:** 10.1001/jamanetworkopen.2021.0037

**Published:** 2021-02-24

**Authors:** Dan Su, Bin Wu, Lizheng Shi

**Affiliations:** 1 Department of Pharmacy, The First Affiliated Hospital of University of Science and Technology of China, Hefei, Anhui, China; 2 Medical Decision and Economic Group, Department of Pharmacy, Ren Ji Hospital, South Campus, Shanghai Jiaotong University School of Medicine, Shanghai, China; 3 Department of Global Health Management and Policy, Tulane University School of Public Health and Tropical Medicine, New Orleans, Louisiana

## Abstract

**Importance:**

Treatment with atezolizumab plus bevacizumab may prolong overall survival among patients with unresectable hepatocellular carcinoma. However, to our knowledge, the cost-effectiveness of using this high-priced therapy for this indication is currently unknown.

**Objective:**

To evaluate the cost-effectiveness of atezolizumab plus bevacizumab to treat unresectable hepatocellular carcinoma from the US payer perspective.

**Design, Setting, and Participants:**

This economic evaluation used a partitioned survival model consisting of 3 discrete health states to assess the cost-effectiveness of treatment of hepatocellular carcinoma with atezolizumab plus bevacizumab vs sorafenib. The characteristics of patients in the model were similar to patients in a phase 3, open-label randomized clinical trial (IMbrave150) who had unresectable hepatocellular carcinoma and had not previously received systemic treatment. Key clinical data were generated from the IMbrave150 trial conducted between March 15, 2018, and January 30, 2019, and cost and health preference data were collected from the literature.

**Main Outcomes and Measures:**

Costs, quality-adjusted life-years (QALYs), incremental cost-utility ratios, incremental net health benefits, and incremental net monetary benefits were calculated for the 2 treatment strategies. Subgroup, 1-way sensitivity, and probabilistic sensitivity analyses were performed.

**Results:**

Treatment of hepatocellular carcinoma with atezolizumab plus bevacizumab added 0.530 QALYs and resulted in an incremental cost of $89 807 compared with sorafenib therapy, which had an incremental cost-utility ratio of $169 223 per QALY gained. The incremental net health benefit was −0.068 QALYs, and the incremental net monetary benefit was −$10 202 at a willingness-to-pay threshold of $150 000/QALY. The probabilistic sensitivity analysis indicated that treatment with atezolizumab plus bevacizumab achieved a 35% probability of cost-effectiveness at a threshold of $150 000/QALY. One-way sensitivity analysis revealed that the results were most sensitive to the hazard ratio of overall survival. The subgroup analysis found that treatment with atezolizumab plus bevacizumab was associated with preferred incremental net health benefits in several subgroups, including patients with hepatitis B and C.

**Conclusions and Relevance:**

Atezolizumab plus bevacizumab treatment is unlikely to be a cost-effective option compared with sorafenib for patients with unresectable hepatocellular carcinoma. Reducing the prices of atezolizumab and bevacizumab may improve cost-effectiveness. The economic outcomes also may be improved by tailoring treatments based on individual patient factors.

## Key Points

**Question:**

Compared with sorafenib, is atezolizumab plus bevacizumab cost-effective as first-line treatment of unresectable hepatocellular carcinoma?

**Findings:**

In this economic evaluation using a partitioned survival model, therapy with atezolizumab plus bevacizumab generated incremental benefit over sorafenib as measured by quality-adjusted life-years but was not cost-effective at a willingness-to-pay threshold of $150 000 per quality-adjusted life-year. However, some patients may achieve preferred economic outcomes from atezolizumab plus bevacizumab therapy by tailoring the regimen based on individual patient factors.

**Meaning:**

The findings suggest that atezolizumab plus bevacizumab may be a valuable therapy for unresectable hepatocellular carcinoma but may become more cost-effective with price reductions.

## Teaser

This economic evaluation study using a partitioned survival model assesses whether atezolizumab plus bevacizumab is cost-effective compared with sorafenib for first-line treatment of unresectable hepatocellular carcinoma.

## Introduction

Hepatocellular carcinoma (HCC) is the third leading cause of cancer death worldwide, accounting for 8.9% of the disease burden of all neoplasms.^1^ This statistic occurs partly because only 30% to 40% of all patients receive a diagnosis at early stages that are amenable to potentially curative treatments.^2^ For more than a decade, the availability of new agents, such as lenvatinib- and sorafenib-based targeted therapy, has significantly improved the outcomes of patients with advanced HCC, increasing the median overall survival (OS) to 10 to 15 months.^3,4^ However, the therapeutic options for HCC are still very limited, and the prognosis is poor.

The open-label, phase 3 randomized clinical trial IMbrave150 conducted between March 15, 2018, and January 30, 2019, reported the efficacy and safety of atezolizumab plus bevacizumab compared with sorafenib for treatment of advanced metastatic or unresectable HCC.^5^ The results revealed that atezolizumab plus bevacizumab markedly prolonged the median progression-free survival (PFS) in comparison with sorafenib (6.8 months vs 4.3 months; hazard ratio [HR], 0.59; 95% CI, 0.47-0.76), and greater OS at 12 months was observed (67.2% vs 54.6%; HR, 0.58 [95% CI, 0.42-0.79]; *P* < .001). The rate of grade 3 or higher adverse drug events was comparable between the 2 groups (56.5% vs 55.1%). Thus, the atezolizumab plus bevacizumab regimen seemed to be an attractive alternative for the treatment of advanced HCC as a first-line option. However, considering cost-effectiveness in health decisions is helpful for clinicians and decision-makers to optimally allocate limited health resources. The present analysis investigated the cost-effectiveness of atezolizumab plus bevacizumab as a first-line therapy for advanced HCC from the US payer perspective.

## Methods

### Analytical Overview

**Figure 1.**
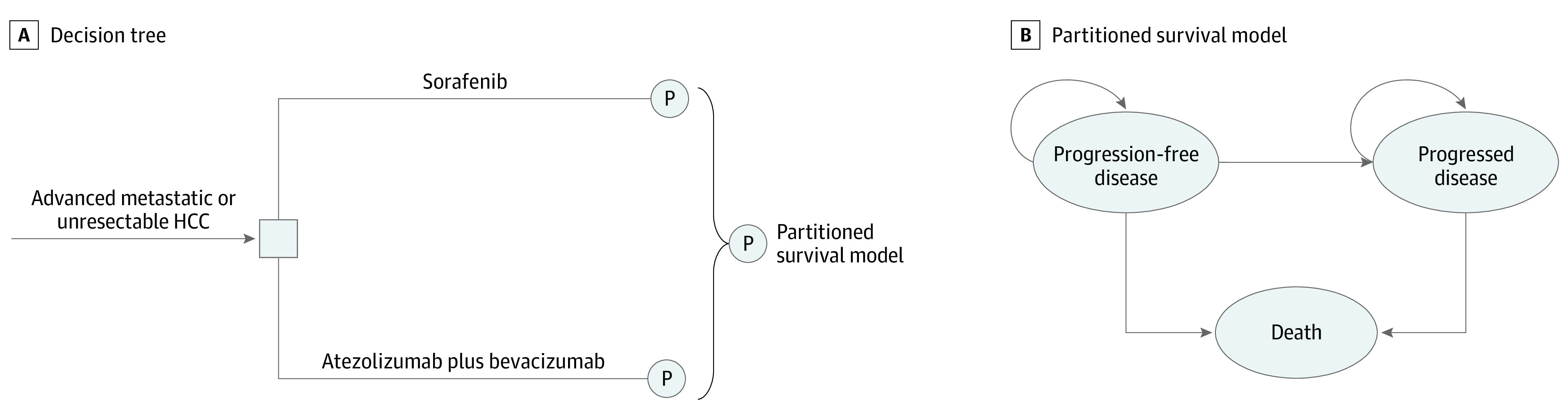
Model Structure of a Decision Tree Combining the Partitioned Survival Model With the 3 Health States HCC indicates hepatocellular carcinoma; P, partitioned survival model.

The hypothetical target population for this analysis was patients who had advanced metastatic or unresectable HCC and did not previously receive systemic treatment, consistent with the patient characteristics of the IMbrave150 trial.^5^ A partitioned survival model with 3 health states was constructed for an initial decision regarding therapy with atezolizumab plus bevacizumab or with sorafenib in this economic evaluation.^6^ As shown in Figure 1, the 3 mutually exclusive health states were progression-free disease (PFD), progressed disease (PD), and death. In the 3 health states, OS was partitioned into alive with PFS and alive and with PD. The proportion of patients alive at cycle *t* (1-week cycle) was estimated by the area under the OS curve, and the proportion alive with PFS was estimated by the area under the PFS curve. The proportion alive and with PD was estimated by the difference between the OS and PFS curves. The proportions of patients with PFS and OS were based on the results of the IMbrave150 trial,^5^ which was validated by comparing modeled PFS and OS results with the observed data. This study followed the Consolidated Health Economic Evaluation Reporting Standards (CHEERS) reporting guideline (eTable 1 in the Supplement).^7^ The First Affiliated Hospital of University of Science and Technology of China declared this study exempt from requiring institutional review board review and from obtaining informed consent because this study was based on a literature review of publicly available data and on modeling techniques.

Because the data maturity of OS was lower than 40% (96 deaths among 336 patients [28.6%] in the atezolizumab plus bevacizumab group and 65 deaths among 165 patients [39.4%] in the sorafenib group) in the IMbrave150 trial, which reported survival data from only 0 to 16 months (median duration of follow-up, 8.1 months),^5^ the present analysis adjusted the OS distribution by using data after 16 months from the open-label, phase 3 randomized clinical trial conducted by Kudo and colleagues.^4^ That trial reported the mature OS data associated with sorafenib treatment for patients with unresectable HCC (median duration of follow-up, 27.2 months), and the median OS time associated with sorafenib was 12.3 months (95% CI, 10.4-13.9 months), which is comparable to that in the sorafenib group of the IMbrave150 trial. Therefore, OS in the sorafenib group from the 17th month to the termination of the model was bridged by OS data from the sorafenib group in the trial by Kudo et al.^4^ The OS of atezolizumab plus bevacizumab from the 17th month to the termination of the model was estimated by multiplying the reported OS rate of sorafenib in that trial and the HR for the OS in the atezolizumab plus bevacizumab group against the sorafenib group in the IMbrave150 trial.

### Clinical Data Inputs

The PFS and OS of patients in the atezolizumab plus bevacizumab group and in the sorafenib group were informed by the results of the IMbrave150 trial^5^ (at least the trial follow-up) and extrapolated beyond the model time horizon using standard statistical analyses described by Guyot et al.^8^ GetData Graph Digitizer, version 2.26,^9^ was used to gather the data points from the PFS and OS curves, and these data points were then used to fit the following parametric survival functions: Weibull, log-normal, log-logistic, exponential, generalized gamma, Gompertz, and Royston-Parmar spline models and parametric mixture and nonmixture cure models. The eligible survival function was chosen based on the lowest value of the Akaike information criterion. The final survival functions of the sorafenib treatment and of the atezolizumab plus bevacizumab treatment are shown in Table 1,^4,5,10,11,12,13,14,15^ and the goodness-of-fit results are shown in eTable 2 in the Supplement. The OS function of sorafenib in the trial by Kudo et al^4^ was independently modeled by using the aforementioned techniques. The proportions of patients with PFS and OS were calculated by using the selected survival distribution. The validation plot, survival distribution, and HRs of the subgroups are shown in eFigures 1, 2, and 3 in the Supplement. Virtual patient-level data comprised event and censor times and were equal in number to the initial number at risk, which closely reproduced the digitized Kaplan-Meier curves. After disease progressed, data from patients who received subsequent treatment were collected from the IMbrave150 trial.^5^ The key clinical inputs are given in Table 1.

**Table 1.** Key Model Inputs

Parameter	Expected value (range)	Distribution	Reference
Clinical input			
Survival model for sorafenib			5
Log-normal model for PFS^a^	Log-mean = 1.9445, log-SD = 1.1894		
Log-logistic model for OS^a^	Shape = 1.5145, rate = 18.5865		
Survival model for atezolizumab plus bevacizumab			5
Royston-Parmar spline model for PFS^a^	Gamma0 = −2.9276, gamma1 = 3.9106, gamma2 = 1.0361, gamma3 = −0.8589		
Log-normal model for OS^a^	Log-mean = 2.5567, log-SD = 1.2314		
Royston-Parmar spline model for OS associated with sorafenib^a^	Gamma0 = −8.7209522, gamma1 = 2.4499163, gamma2 = 0.1511148		4
HR for PFS associated with atezolizumab plus bevacizumab vs sorafenib	0.59 (0.47 to 0.76)	Log-normal: log-mean = −0.528, log-SD = 2.604	5
HR for OS associated with atezolizumab plus bevacizumab vs sorafenib	0.58 (0.42 to 0.79)	Log-normal: log-mean = −0.545, log-SD = 2.36	5
Proportion receiving subsequent treatment			5
Sorafenib	0.67 (0.50 to 0.84)	Beta: α = 5.3, β = 2.6	
Atezolizumab plus bevacizumab	0.35 (0.26 to 0.44)	Beta: α = 10.4, β = 19.3	
Utility input			
Utility of PFD	0.76 (0.61 to 0.91)	Beta: α = 4.7, β = 1.5	10
Utility of PD	0.68 (0.54 to 0.82)	Beta: α = 29, β = 13.6	10
Disutility due to AEs			
Grade 1 and 2	0.01 (0.008 to 0.020)	Beta: α = 18, β = 1283.2	11
Grade 3 and higher	0.16 (0.110 to 0.204)	Beta: α = 36, β = 193	11
Cost input			
Atezolizumab per 1200 mg^b^	9280 (4640 to 9280)	Fixed	12, 13
Bevacizumab per 100 mg^b^	841 (420 to 841)	Fixed	12, 13
Sorafenib per 200 mg^c^	174 (87 to 174)	Fixed	12, 13
Subsequent active treatment per patient^d^	108 336 (81 252 to 135 420)	Gamma: α = 433 345, λ = 0.25	14
Subsequent best supportive care per patient^d^	37 084 (27 813 to 46 355)	Gamma: α = 148 337, λ = 0.25	14
Follow-up and monitoring per month			
Patients with PFD^e^	787 (590 to 984)	Gamma: α = 3149, λ = 0.25	15
Patients with PD^e^	915 (686 to 1144)	Gamma: α = 3659, λ = 0.25	15
Drug administration per unit	298 (223 to 372)	Gamma: α = 1191, λ = 0.25	11
Terminal care per patient^d^	7894 (6315 to 9473)	Gamma: α = 77 390, λ = 0.102	14

Abbreviations: AE, adverse event; HR, hazard ratio; OS, overall survival; PD, progressed disease; PFD, progression-free disease; PFS, progression-free survival.

^a^ Only expected values are presented for these survival model parameters.

^b^ Treatment with atezolizumab plus bevacizumab continued until disease progression, unacceptable toxicity, or 2 years of follow-up.

^c^ Treatment with sorafenib continued until disease progression or unacceptable toxicity.

^d^ Overall total cost per patient regardless of treatment duration.

^e^ These costs were assumed to be continued until the health state transitioned.

### Cost and Utility Inputs

Only direct medical costs, including costs of acquiring drugs, costs attributed to the patient’s health state, costs for the management of adverse events (AEs), and costs for end-of-life care, were analyzed (Table 1). The costs are reported in 2019 US dollars and were inflated to 2019 values using the Medical-Care Inflation data set in Tom’s Inflation Calculator.^16^

According to the IMbrave150 trial report,^5^ patients in the atezolizumab plus bevacizumab group received atezolizumab (1200 mg) plus bevacizumab (15 mg/kg body weight) intravenously every 3 weeks. Patients assigned to the sorafenib group received sorafenib (400 mg) orally twice daily. Treatment continued until disease progression or unacceptable toxicity or, for the immunotherapy regimen group, until 2 years of follow-up. The prices of atezolizumab, bevacizumab, and sorafenib were collected from public databases.^12,13^ In the US, the prices of ipilimumab, nivolumab, pembrolizumab, and dabrafenib plus trametinib were discounted by 17% to account for contract pricing.^17^ To calculate the dosage of bevacizumab, we assumed that a typical patient in the US weighed 71.4 kg.^18^ After disease progression, 69 of 197 patients (35.0%) in the atezolizumab plus bevacizumab group and 73 of 109 patients (67.0%) in the sorafenib group received subsequent active therapy. The costs associated with subsequent active salvage therapy and the greatest supportive care were $108 336 and $37 084 per patient, respectively, which were estimated from a cost-effectiveness analysis of second-line treatments of advanced HCC.^14^ The monitoring costs for patients with PFD and patients with PD were $245 per month and $15 308 per month, respectively, which were collected from an economic evaluation of sorafenib for unresectable HCC.^15^ The cost associated with terminal care was $7893 per patient with advanced HCC.^14^ The analysis included the costs associated with managing grade 3 or higher AEs, which were extracted from the literature (eTable 3 in the Supplement).^14,19^

Each health state was assigned a health utility preference on a scale of 0 (death) to 1 (perfect health). The PFD and PD states associated with HCC were 0.76 and 0.68,^10^ respectively, which were derived from a cost-effectiveness analysis considering patients with HCC. The disutility values due to grade 1 or 2 and grade 3 or 4 AEs were included in this analysis.^11^ All AEs were assumed to be incurred during the first cycle. The duration-adjusted disutility was subtracted from the baseline PFD utility.

#### Base-Case Analysis

The incremental cost-utility ratio (ICUR) was calculated as the incremental cost per additional quality-adjusted life-year (QALY) gained between the atezolizumab plus bevacizumab group and the sorafenib group. When the ICUR was lower than the prespecified willingness-to-pay threshold ($150 000 per additional QALY gained), cost-effectiveness was assumed according to the recommendation.^20^ Costs and QALYs were reduced at an annual rate of 3%.^21^ We also estimated the incremental net health benefit (INHB) and incremental monetary benefit (INMB) based on the following formulas: INHB(λ) = (μ*_E_*_1_ − μ*_E_*_0_) − (μ*_C_*_1_ − μ*_C_*_0_)/λ = Δ*E* − Δ*C*/λ and INMB(λ) = (μ*_E_*_1_ − μ*_E_*_0_) × λ − (μ*_C_*_1_ − μ*_C_*_0_) = Δ*E* × λ − Δ*C*, where μ*_Ci_* and μ*_Ei_* were the cost and effectiveness of atezolizumab plus bevacizumab (*i* = 1) or sorafenib (*i* = 0), respectively, and λ was the willingness-to-pay threshold.^22,23^

#### Sensitivity and Subgroup Analyses

To evaluate the robustness of the base-case result, we conducted 1-way sensitivity analyses and probabilistic sensitivity analyses. One-way sensitivity analyses were conducted for all parameters, and the estimated range of each parameter was based on either the reported or estimated 95% CIs in the referenced studies or determined by assuming a 25% change from the base-case value (Table 1). In the probabilistic sensitivity analysis, a Monte Carlo simulation with 10 000 iterations was generated by simultaneously sampling the key model parameters from the prespecified distributions. A gamma distribution was selected for the cost parameters, a log-normal distribution for the HRs, and a beta distribution for probability, proportion, and preference value parameters. Based on the data from 10 000 iterations, a cost-effectiveness acceptability curve was created to represent the likelihood that atezolizumab plus bevacizumab would be considered cost-effective at various willingness-to-pay levels for health gains (QALYs). To investigate the uncertainty of economic outcomes caused by the subpopulations, exploratory subgroup analyses were performed for the prespecified subgroups that were reported in the IMbrave150 trial by varying the HRs for PFS and OS. Programming and statistical analyses were conducted with hesim and heemod packages in R, version 3.5.3, 2019 (R Foundation for Statistical Computing).

## Results

### Base-Case Analysis

In comparison with sorafenib therapy, atezolizumab plus bevacizumab treatment provided an additional 0.530 QALYs and 1.297 overall life-years, with an incremental cost of $89 807, which was associated with an ICUR of $169 223/QALY. The INHB was −0.068 QALYs, and the INMB was −$10 202 at a willingness-to-pay threshold of $150 000/QALY (Table 2).

**Table 2.** Summary of Cost and Outcome Results in the Base-Case Analysis

Strategy	Sorafenib	Atezolizumab plus bevacizumab
Cost, $	202 973	292 780
First-line drug	109 355	214 210
Other	93 619	78 570
Life-years		
Progression-free	0.548	0.938
Overall	1.736	3.033
QALYs	1.021	1.551
Incremental cost per QALY^a^	NA	169 223
INHB, QALY^a^	NA	−0.068
INMB, $^a^	NA	−10 202

Abbreviations: INHB, incremental net health benefit; INMB, incremental net monetary benefit; NA, not applicable; QALY, quality-adjusted life-years.

^a^ Compared with sorafenib.

### Sensitivity Analysis

One-way sensitivity analyses revealed that the HRs for OS and the costs of bevacizumab, sorafenib, and atezolizumab were associated with model outcomes (eFigure 4 in the Supplement). When the lower boundary of the HR (ie, 0.42) for OS was adopted, the ICUR of atezolizumab plus bevacizumab vs sorafenib was $109 143 per additional QALY gained, and when the upper boundary (HR = 0.79) was adopted, the ICUR was $303 346 per additional QALY gained. When the cost of atezolizumab and bevacizumab was discounted by 50%, the ICUR was less than $100 000 per additional QALY. The low cost of sorafenib, high body weight, and HRs for PFS were associated with ICURs exceeding the threshold of $200 000/QALY. The remaining parameters, such as the cost and utility related to AEs, had only moderate or low associations with the outcome and were not associated with ICURs exceeding the threshold.

**Figure 2.**
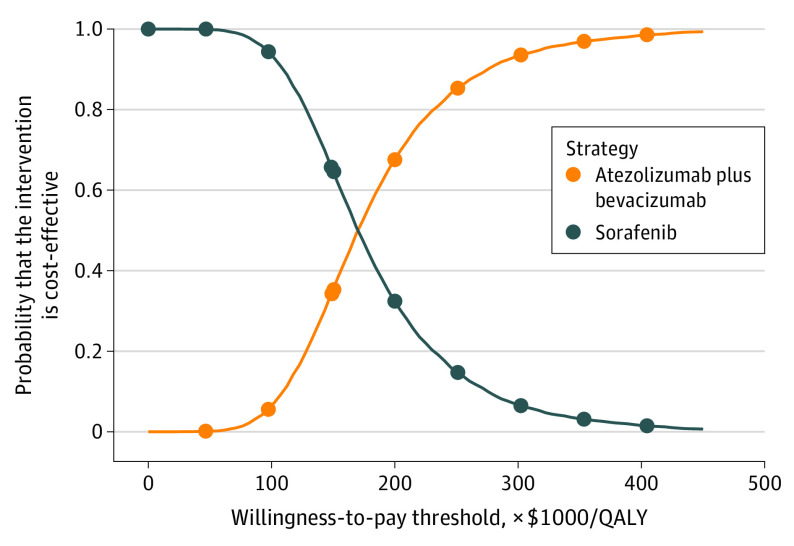
Cost-effectiveness Acceptability Curves for Atezolizumab Plus Bevacizumab vs Sorafenib QALY indicates quality-adjusted life-year.

Compared with sorafenib, the probabilistic sensitivity analysis showed that atezolizumab plus bevacizumab added a mean of 0.539 QALYs (range of 95% of all values, 0.277-0.842) with an additional mean cost of $91 512 (range of 95% of all values, $53 413-$131 104), which resulted in a mean ICUR of $184 257/QALY (range of 95% of all values, $83 788/QALY-$370 669/QALY). The cost-effectiveness acceptability curve showed that the probability of atezolizumab plus bevacizumab being cost-effective increased from 35% to 68% when the threshold value ranged from $150 000/QALY to $200 000/QALY (Figure 2).

### Subgroup Analysis

**Figure 3.**
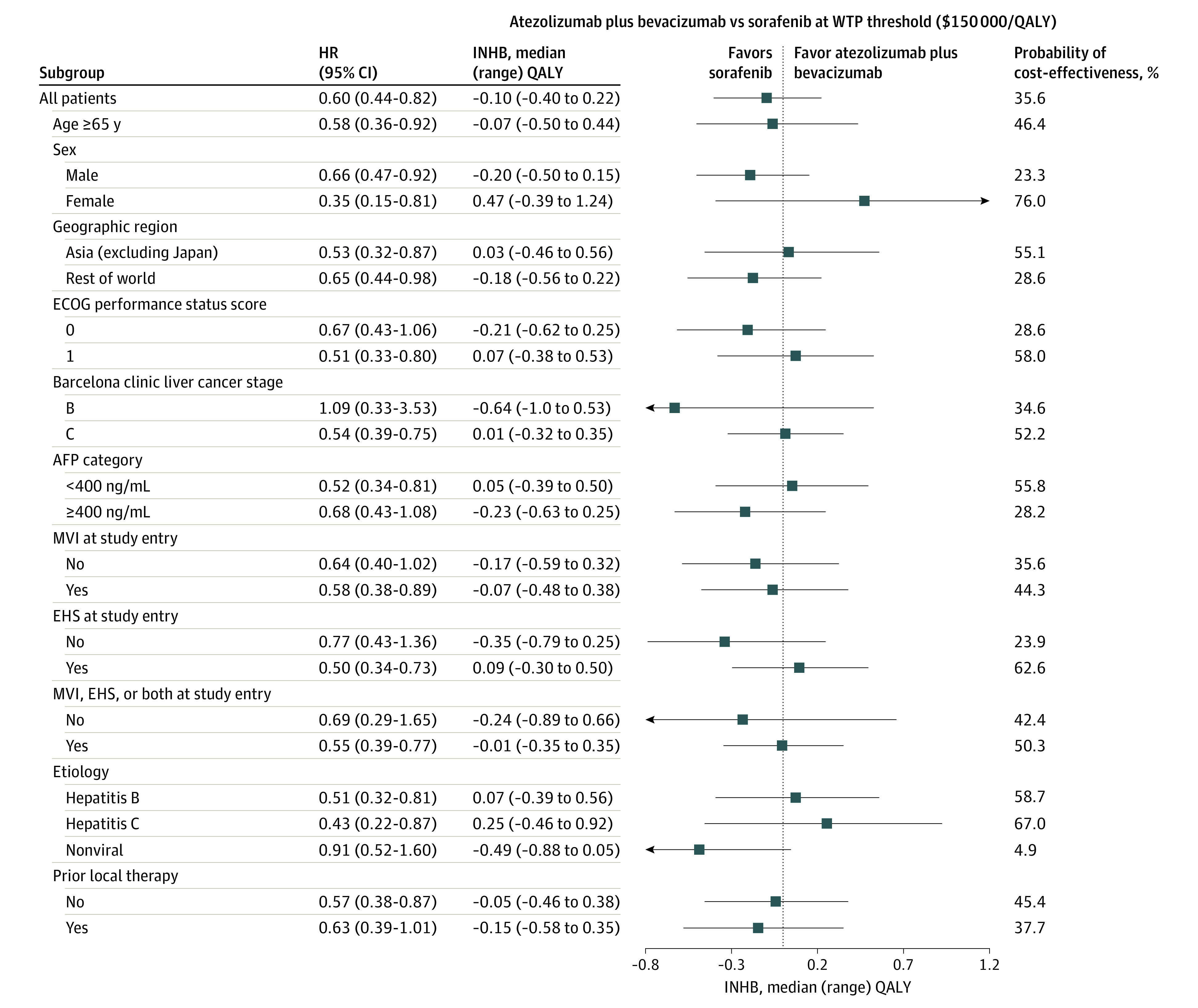
Subgroup Analysis Results of Incremental Net Health Benefits (INHBs) and Probabilities of Cost-effectiveness Obtained by Varying the Hazard Ratios (HRs) for Overall Survival AFP indicates α-fetoprotein (to convert α-fetoprotein level to micrograms per liter, multiply by 1.0); EHS, represents extrahepatic spread; ECOG, Eastern Cooperative Oncology Group; MVI, macrovascular invasion; QALY, quality-adjusted life-year; and WTP, willingness to pay. Vertical line indicates the point of no association (INHB, 0); squares, median INHB; and horizontal lines, ranges of INHBs adjusted by HRs.

The subgroup analyses, which were conducted by varying the HRs for OS, revealed that atezolizumab plus bevacizumab was associated with primarily positive INHBs and greater than 50% probability of being cost-effective in the following subgroups at the threshold of $150 000/QALY (Figure 3): female patients, patients who lived in Asia (excluding Japan), patients with an Eastern Cooperative Oncology Group score of 1, patients with Barcelona clinic liver cancer stage C disease, patients with an α-fetoprotein level lower than 400 ng/mL (to convert α-fetoprotein level to micrograms per liter, multiply by 1.0), patients with extrahepatic spread at study entry, and patients with HCC caused by hepatitis B or C. The INHBs in the subgroups varied from −0.49 (range, −0.88 to 0.05; probability of cost-effectiveness, 4.9%) for patients with nonviral HCC to 0.47 (range, −0.39 to 1.24; probability of cost-effectiveness, 76.0%) for female patients. The subgroup analyses performed by varying the HRs for PFS found that atezolizumab plus bevacizumab was associated with primarily negative INHBs, and the probability of cost-effectiveness was lower than 50% in most of the subgroups (eFigure 5 in the Supplement).

## Discussion

Our study addresses the unmet need for an economic assessment of atezolizumab plus bevacizumab. Based on the results of the IMbrave150 trial, our analysis showed that atezolizumab plus bevacizumab for treatment of advanced HCC was unfavorable for willingness-to-pay thresholds lower than $169 223 per QALY. This finding is generally robust, as shown by the results of the probabilistic sensitivity analysis. At a threshold of $150 000/QALY, 7 subgroups, including Eastern Cooperative Oncology Group scores of 1 and HCC associated with hepatitis B or C, favored atezolizumab plus bevacizumab treatment owing to its association with positive INHBs and higher than 50% probability of cost-effectiveness compared with sorafenib. In the recent letter reported by Hou and Wu,^24^ the cost-effectiveness of atezolizumab plus bevacizumab vs sorafenib was also investigated among patients in China. That study gained incremental survival benefits similar to those in our study because the same trial data and survival curve simulation technique were adopted. Although they used local-specific cost inputs and a discount rate, which was associated with different incremental costs and QALYs than ours, atezolizumab plus bevacizumab was also found to not be a cost-effective option.

The ability of atezolizumab plus bevacizumab to prevent disease-related death was a major factor associated with economic outcomes. The findings of the 1-way sensitivity analysis showed that the HR for OS was the most sensitive parameter. This result indicated that atezolizumab plus bevacizumab was more cost-effective for patients with a favorable prognosis, such as female patients and patients with HCC caused by hepatitis B or C infection, than for patients with a poor prognosis. However, for patients with an unfavorable HR for OS who had a high risk of mortality, such as the subgroup with Barcelona clinic liver cancer stage B or nonviral HCC, atezolizumab plus bevacizumab treatment may not be cost-effective. The costs of bevacizumab, sorafenib, and atezolizumab were also found to be important. When the costs of bevacizumab and atezolizumab were reduced by 50%, atezolizumab plus bevacizumab treatment became favorable because its ICUR was lower than $100 000/QALY. Recently, the US government has proposed indexing the prices that Medicare pays for drugs to those paid by health systems in other developed countries to help reduce the relatively high prices paid by US patients.^25^ Once this indexing system is enacted or implemented, the initiative may lead to a reduction in the price of atezolizumab, which may result in more favorable economic outcomes.

### Strengths and Limitations

The strengths of this study are worth highlighting. First, to our knowledge, this is the first analysis to simultaneously evaluate the economic outcomes of atezolizumab plus bevacizumab treatment of unresectable HCC by synthesizing the latest evidence through an economic modeling approach. Monotherapy blockade of programmed cell death 1 alone or in combination with other regimens is becoming a popular choice for the treatment of advanced HCC.^26^ However, to our knowledge, data on the economic outcomes of immune checkpoint inhibitors (ICIs) for the treatment of advanced HCC are scarce. Second, the present analysis examined the economic outcomes of 22 subgroups prespecified by the IMbrave150 trial. Economic information for the subgroups may help tailor treatment decisions of physicians, patients, and policy makers. Further work needs to confirm who may or may not benefit from treatment with atezolizumab plus bevacizumab.

There are several limitations in the analysis. First, owing to the lack of head-to-head data, we did not include other ICIs as first-line treatments, such as pembrolizumab and nivolumab, which have shown favorable health benefits for patients with advanced HCC as second-line strategies.^26^ The present analysis should be updated when first-line data become available. Second, health benefits beyond the observation time of the IMbrave150 trial were assumed through the fitting of parametric distributions to the reported Kaplan-Meier PFS and OS data, which may result in uncertainty in the model outputs, although the modeled and observed data were validated. Third, we did not measure the economic changes in society associated with adding atezolizumab. Because 7500 new patients with HCC and late-stage disease would be eligible for approximately 10 first-line treatment cycles of ICI treatments annually,^5,27^ first-line prescription of atezolizumab plus bevacizumab may markedly increase the financial burden. Fourth, owing to the absence of time series data, the present analysis did not consider the associations of the cost varied by survival time and duration, such as costs associated with follow-up. Our sensitivity analysis showed that cost inputs were not associated with the model outputs except for the costs of bevacizumab, sorafenib, and atezolizumab. Fifth, costs of grade 1 or 2 AEs were excluded from the evaluation, which may have overestimated the economic results associated with atezolizumab plus bevacizumab. This limitation may not be a major factor, as suggested by the findings in the 1-way sensitivity analysis indicating that the costs associated with AEs were minor. However, because the findings of this evaluation reflected general clinical practice of managing advanced HCC, they may be a valuable reference for physicians and policy makers.

## Conclusions

These estimates showed that atezolizumab plus bevacizumab was unlikely to be a cost-effective first-line option for patients with unresectable HCC. The economic outcomes may be improved by tailoring treatments based on individual patient factors. Reducing the cost of bevacizumab and atezolizumab may yield favorable economic outcomes. These findings may aid clinicians in making optimal decisions regarding the treatment of advanced HCC. Because of method limitations in the present study, additional high-quality clinical and economic real-world data are needed; we believe that this focus will provide sound evidence to serve as a framework for determining the value of different therapeutic alternatives in oncology.

## Notes

Supplement. **eFigure 1.** The Replicated Kaplan-Meier PFS Curves of Atezolizumab Plus Bevacizumab and Sorafenib in the IMbrave150 Trial **eFigure 2.** The Replicated Kaplan-Meier OS Curves of Atezolizumab Plus Bevacizumab and Sorafenib in the IMbrave150 Trial **eFigure 3.** The Replicated Kaplan-Meier OS Curves of Lenvatinib and Sorafenib in the Kudo and Colleague’s Trial **eFigure 4.** Tornado Diagram of 1-Way Sensitivity Analyses of Atezolizumab Plus Bevacizumab Versus Sorafenib in Order of Magnitude of the Association **eFigure 5.** Subgroup Analysis of Incremental Net Health Benefits (INHB) and Probabilities of Cost-effectiveness by Varying the Hazard Ratios (HRs) of PFS **eTable 1.** CHEERS Checklist **eTable 2.** Estimated Parameters and AIC Values From Each Survival Model **eTable 3.** Probability and Costs Related to Adverse Events (Grade ≥3)
