# Supplementary material for: Cost-effectiveness of Atezolizumab Plus Bevacizumab vs Sorafenib as First-Line Treatment of Unresectable Hepatocellular Carcinoma
Source: JAMA Netw Open. 2021 Feb 24;4(2):e210037. doi: 10.1001/jamanetworkopen.2021.0037 (PMC7905498; doi:10.1001/jamanetworkopen.2021.0037)
Supplement: Supplement. — eFigure 1. The Replicated Kaplan-Meier PFS Curves of Atezolizumab Plus Bevacizumab and Sorafenib in the IMbrave150 Trial eFigure 2. The Replicated Kaplan-Meier OS Curves of Atezolizumab Plus Bevacizumab and Sorafenib in the IMbrave150 Trial eFigure 3. The Replicated Kaplan-Meier OS Curves of Lenvatinib and Sorafenib in the Kudo and Colleague’s Trial eFigure 4. Tornado Diagram of 1-Way Sensitivity Analyses of Atezolizumab Plus Bevacizumab Versus Sorafenib in Order of Magnitude of the Association eFigure 5. Subgroup Analysis of Incremental Net Health Benefits (INHB) and Probabilities of Cost-effectiveness by Varying the Hazard Ratios (HRs) of PFS eTable 1. CHEERS Checklist eTable 2. Estimated Parameters and AIC Values From Each Survival Model eTable 3. Probability and Costs Related to Adverse Events (Grade ≥3) [file jamanetwopen-e210037-s001.pdf]

Supplementary Online Content

Su D, Wu B, Shi L. Cost-effectiveness of atezolizumab plus bevacizumab vs sorafenib as first-line treatment of unresectable hepatocellular carcinoma. *JAMA Netw Open*. 2021;4(2):e210037. doi:10.1001/jamanetworkopen.2021.0037

eFigure 1. The Replicated Kaplan-Meier PFS Curves of Atezolizumab Plus Bevacizumab and Sorafenib in the IMbrave150 Trial

eFigure 2. The Replicated Kaplan-Meier OS Curves of Atezolizumab Plus Bevacizumab and Sorafenib in the IMbrave150 Trial

eFigure 3. The Replicated Kaplan-Meier OS Curves of Lenvatinib and Sorafenib in the Kudo and Colleague's Trial

eFigure 4. Tornado Diagram of 1-Way Sensitivity Analyses of Atezolizumab Plus Bevacizumab Versus Sorafenib in Order of Magnitude of the Association

eFigure 5. Subgroup Analysis of Incremental Net Health Benefits (INHB) and Probabilities of Cost-effectiveness by Varying the Hazard Ratios (HRs) of PFS

eTable 1. CHEERS Checklist

eTable 2. Estimated Parameters and AIC Values From Each Survival Model

eTable 3. Probability and Costs Related to Adverse Events (Grade ≥3)

This supplementary material has been provided by the authors to give readers additional information about their work.

© 2021 Su D et al. *JAMA Network Open*.

eFigure 1. The Replicated Kaplan-Meier PFS Curves of Atezolizumab Plus Bevacizumab and Sorafenib in the IMbrave150 Trial

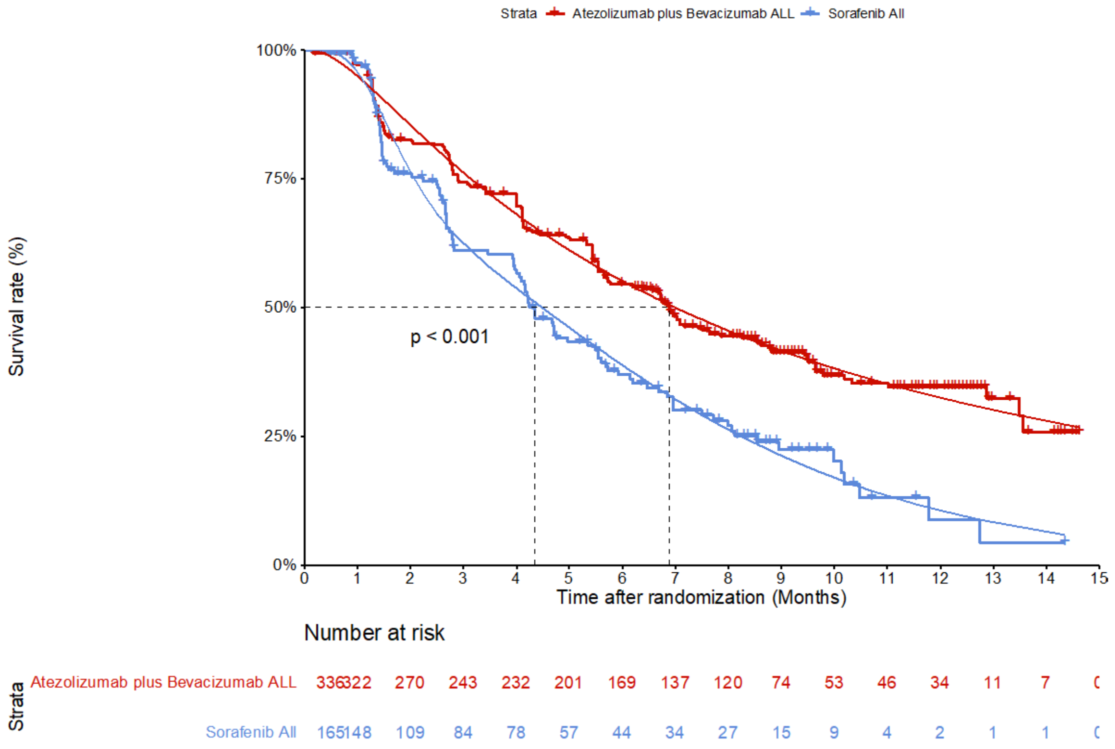

The smooth lines indicated the survival curves predicting their corresponding best survival distributions.

© 2021 Su D et al. *JAMA Network Open*.

eFigure 2. The Replicated Kaplan-Meier OS Curves of Atezolizumab Plus Bevacizumab and Sorafenib in the IMbrave150 Trial

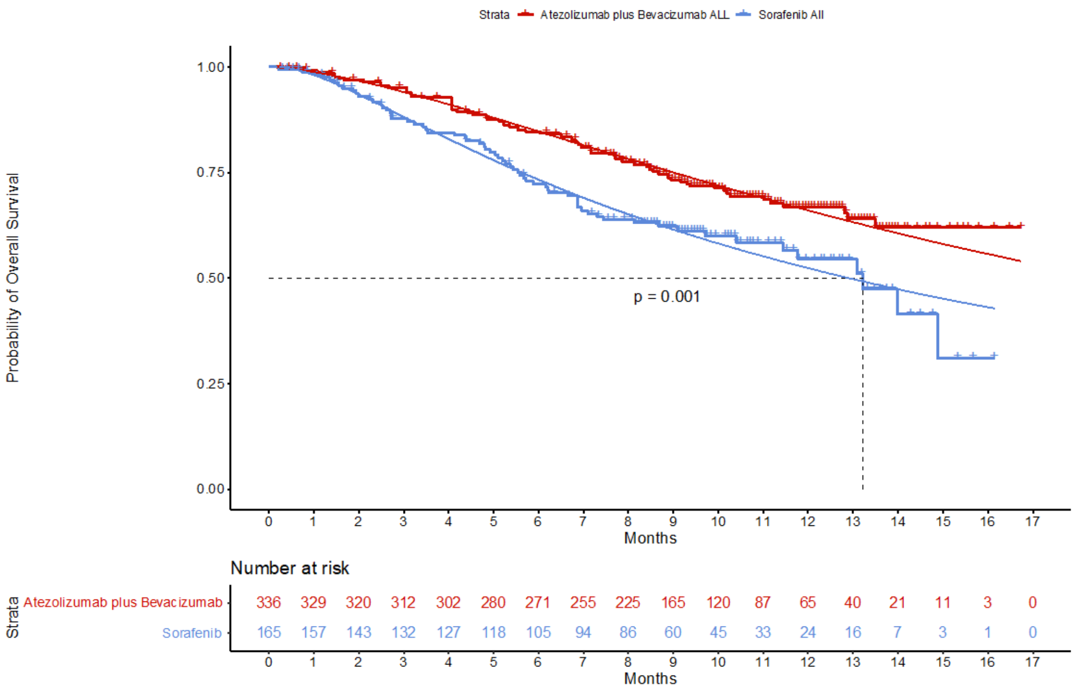

The smooth lines indicated the survival curves predicting their corresponding best survival distributions.

© 2021 Su D et al. *JAMA Network Open*.

eFigure 3. The Replicated Kaplan-Meier OS Curves of Lenvatinib and Sorafenib in the Kudo and Colleague’s Trial

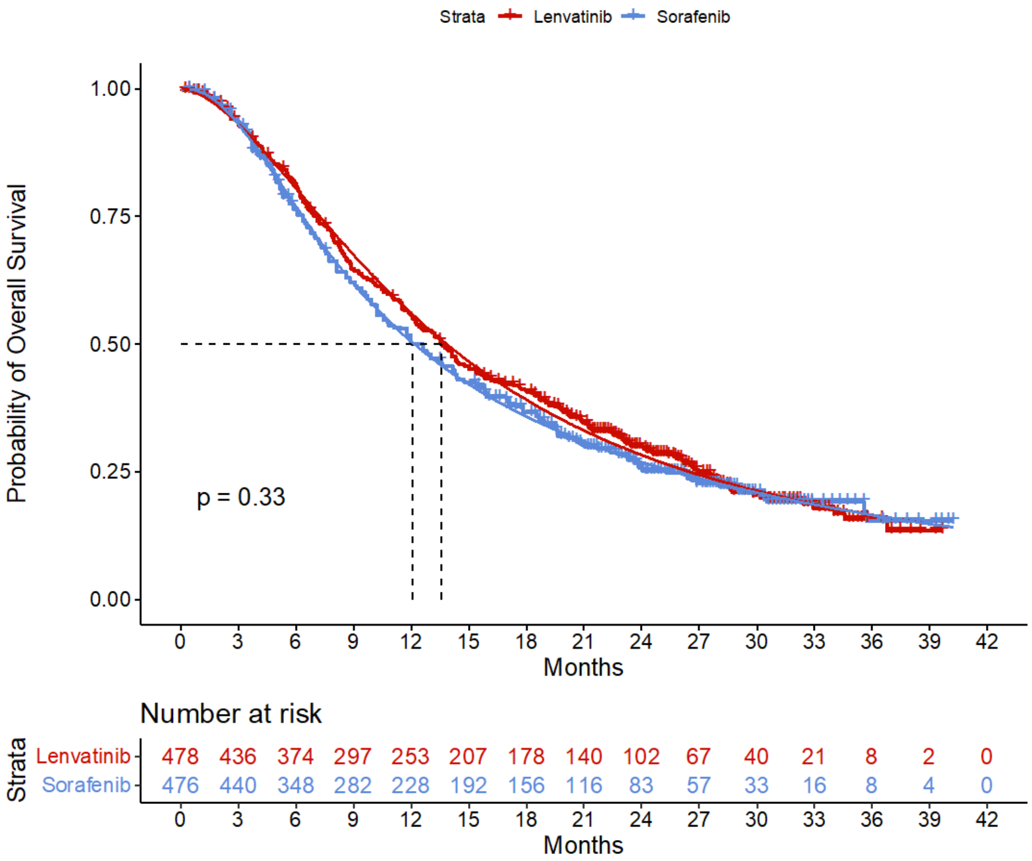

The smooth lines indicated the survival curves predicting their corresponding best survival distributions.

© 2021 Su D et al. *JAMA Network Open*.

eFigure 4. Tornado Diagram of 1-Way Sensitivity Analyses of Atezolizumab Plus Bevacizumab Versus Sorafenib in Order of Magnitude of the Association

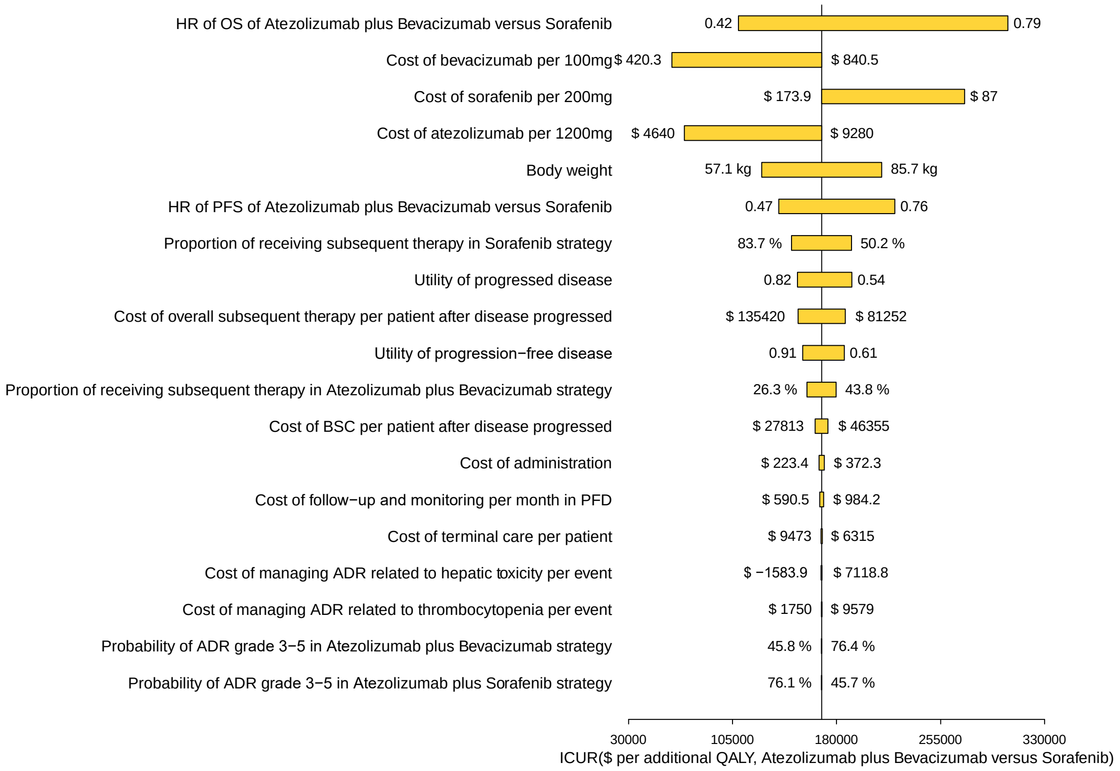

© 2021 Su D et al. *JAMA Network Open*.

eFigure 5. Subgroup Analysis of Incremental Net Health Benefits (INHB) and Probabilities of Cost-effectiveness by Varying the Hazard Ratios (HRs) of PFS

Atezolizumab plus Bevacizumab VS. Sorafenib at WTP threshold ($150,000/QALY)

Subgroup	Hazard ratio (95% CI)	Incremental net health benefits (QALY, median[Range])	Probability of cost−effectiveness
All patients	0.59 (0.47 to 0.75)	−0.1 (−0.22 to 0.05)	17.0%
Age ≥65 years	0.63 (0.45 to 0.89)	−0.06 (−0.25 to 0.15)	38.1%
Sex			
Male	0.59 (0.45 to 0.77)	−0.1 (−0.25 to 0.06)	20.1%
Female	0.6 (0.34 to 1.06)	−0.09 (−0.38 to 0.26)	40.5%
Geographic region			
Asia (excluding Japan)	0.46 (0.31 to 0.67)	−0.23 (−0.42 to −0.02)	0.0%
Rest of world	0.7 (0.52 to 0.96)	0 (−0.17 to 0.2)	54.1%
ECOG performance status score			
0	0.57 (0.42 to 0.78)	−0.12 (−0.28 to 0.07)	19.9%
1	0.63 (0.44 to 0.91)	−0.06 (−0.26 to 0.16)	39.1%
Barcelona Clinic liver cancer stage			
B	0.65 (0.33 to 1.3)	−0.04 (−0.4 to 0.38)	49.3%
C	0.58 (0.45 to 0.75)	−0.11 (−0.25 to 0.05)	15.7%
AFP category			
<400 ng/ml	0.49 (0.36 to 0.66)	−0.2 (−0.36 to −0.03)	0.0%
≥400 ng/ml	0.79 (0.54 to 1.16)	0.08 (−0.15 to 0.32)	68.2%
MVI at study entry			
No	0.65 (0.48 to 0.9)	−0.04 (−0.21 to 0.16)	42.8%
Yes	0.53 (0.37 to 0.76)	−0.16 (−0.34 to 0.05)	13.6%
Extrahepatic spread at study entry			
No	0.61 (0.41 to 0.91)	−0.08 (−0.29 to 0.16)	36.0%
Yes	0.54 (0.4 to 0.73)	−0.15 (−0.3 to 0.03)	8.8%
MVI and/or EHS at study entry			
No	0.72 (0.42 to 1.2)	0.02 (−0.28 to 0.34)	54.5%
Yes	0.53 (0.41 to 0.7)	−0.16 (−0.29 to 0)	1.3%
Etiology			
Hepatitis B	0.47 (0.33 to 0.67)	−0.22 (−0.4 to −0.02)	0.0%
Hepatitis C	0.69 (0.39 to 1.2)	0 (−0.32 to 0.34)	51.4%
Nonviral	0.71 (0.47 to 1.08)	0.01 (−0.22 to 0.27)	54.9%
Prior local therapy			
No	0.68 (0.49 to 0.93)	−0.01 (−0.2 to 0.18)	47.1%
Yes	0.51 (0.35 to 0.72)	−0.18 (−0.37 to 0.02)	5.4%

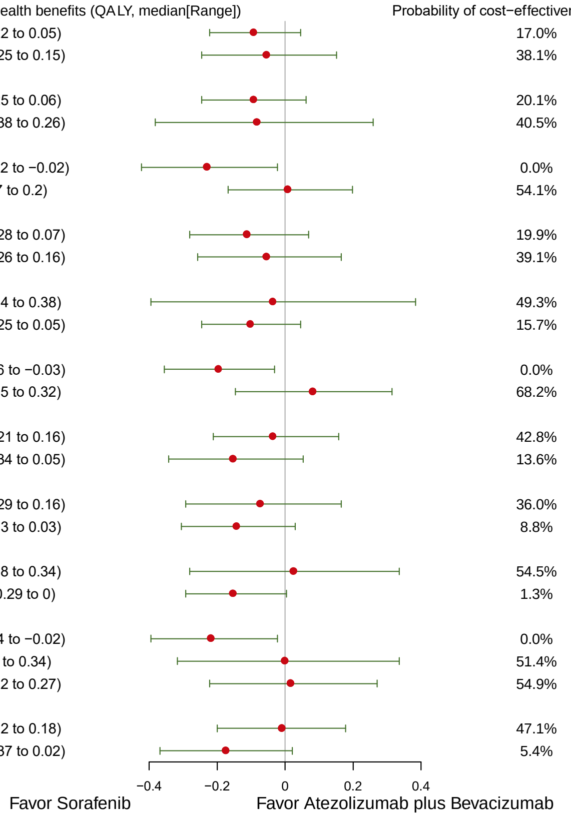

The vertical line indicates the point of no effect (INHB = 0), the red circle indicates the median INHB, and the green bar indicates the ranges of INHB adjusted by the HRs.

© 2021 Su D et al. *JAMA Network Open*.

eTable 1. CHEERS Checklist

Section	Item No	Recommendation	Reported ?
**Title and Abstract**			
Title	1	Identify the study as an economic evaluation or use more specific terms such as “cost-effectiveness analysis”, and describe the interventions compared.	✓
Abstract	2	Provide a structured summary of objectives, perspective, setting, methods (including study design and inputs), results (including base case and uncertainty analyses), and conclusions.	✓
**Introduction**			
Background and objectives	3	Provide an explicit statement of the broader context for the study. Present the study question and its relevance for health policy or practice decisions.	✓
**Methods**			
Target population and subgroups	4	Describe characteristics of the base case population and subgroups analysed, including why they were chosen.	✓
Setting and location	5	State relevant aspects of the system(s) in which the decision(s) need(s) to be made.	✓

© 2021 Su D et al. *JAMA Network Open*.

Study perspective	6	Describe the perspective of the study and relate this to the<br>costs being evaluated.	✓
Comparators	7	Describe the interventions or strategies being compared and<br>state why they were chosen.	✓
Time horizon	8	State the time horizon(s) over which costs and consequences are being evaluated and say why appropriate.	✓
Discount rate	9	Report the choice of discount rate(s) used for costs and<br>outcomes and say why appropriate.	✓
Choice of health outcomes	10	Describe what outcomes were used as the measure(s) of<br>benefit in the evaluation and their relevance for the type of<br>analysis performed.	✓
Measurementof effectiveness	11a	Single study-based estimates: Describe fully the design<br>features of the single effectiveness study and why the single<br>study was a sufficient source of clinical effectiveness data.	NA
	11b	Synthesis-based estimates: Describe fully the methods used for identification of included studies and synthesis of clinical effectiveness data.	✓
Measurement and valuation of preference based outcomes	12	If applicable, describe the population and methods used to<br>elicit preferences for outcomes.	✓

© 2021 Su D et al. *JAMA Network Open*.

Estimating resources and costs	13a	Single study-based economic evaluation: Describe approaches used to estimate resource use associated with the alternative interventions. Describe primary or secondary research methods for valuing each resource item in terms of its unit cost. Describe any adjustments made to approximate to opportunity costs.	NA
	13b	Model-based economic evaluation: Describe approaches and data sources used to estimate resource use associated with model health states. Describe primary or secondary research methods for valuing each resource item in terms of its unit cost. Describe any adjustments made to approximate to opportunity costs.	✓
Currency, price date, and conversion	14	Report the dates of the estimated resource quantities and unit costs. Describe methods for adjusting estimated unit costs to the year of reported costs if necessary. Describe methods for converting costs into a common currency base	✓
		and the exchange rate.	
Choice of model	15	Describe and give reasons for the specific type of decision-analytical model used. Providing a figure to show model structure is strongly recommended.	✓
Assumptions	16	Describe all structural or other assumptions underpinning the decision-analytical model.	✓
Analytical methods	17	Describe all analytical methods supporting the evaluation. This could include methods for dealing with skewed, missing, or censored data; extrapolation methods; methods for pooling	✓

© 2021 Su D et al. *JAMA Network Open*.

		data; approaches to validate or make adjustments (such as half cycle corrections) to a model; and methods for handling population heterogeneity and uncertainty.	
**Results**			
Study parameters	18	Report the values, ranges, references, and, if used, probability distributions for all parameters. Report reasons or sources for distributions used to represent uncertainty where appropriate. Providing a table to show the input values is strongly recommended.	✓
Incremental costs and outcomes	19	For each intervention, report mean values for the main<br>categories of estimated costs and outcomes of interest, as well as mean differences between the comparator groups. If<br>applicable, report incremental cost-effectiveness ratios.	✓
Characterizing uncertainty	20<br>a	Single study-based economic evaluation: Describe the effects of sampling uncertainty for the estimated incremental cost and incremental effectiveness parameters, together with the impact of methodological assumptions (such as discount rate, study perspective).	NA
	20<br>b	Model-based economic evaluation: Describe the effects on the results of uncertainty for all input parameters, and uncertainty related to the structure of the model and assumptions.	✓

© 2021 Su D et al. *JAMA Network Open*.

Characterizing heterogeneity	21	If applicable, report differences in costs, outcomes, or cost-effectiveness that can be explained by variations between subgroups of patients with different baseline characteristics or other observed variability in effects that are not reducible by more information.	✓
**Discussion**			
Study findings, limitations, generalizability, and current knowledge	22	Summarise key study findings and describe how they support the conclusions reached. Discuss limitations and the generalisability of the findings and how the findings fit with current knowledge.	✓
**Other**			
Source of funding	23	Describe how the study was funded and the role of the funder in the identification, design, conduct, and reporting of the analysis. Describe other non-monetary sources of support.	✓
Conflicts of interest	24	Describe any potential for conflict of interest of study contributors in accordance with journal policy. In the absence of a journal policy, we recommend authors comply with International Committee of Medical Journal Editors recommendations.	✓

© 2021 Su D et al. *JAMA Network Open*.

eTable 2. Estimated Parameters and AIC Values From Each Survival Model

Strategies	Distributions	Parameters	PFS					OS				
			est	L95%	U95%	se	AIC	est	L95%	U95%	se	AIC
Sorafenib	Weibull	shape	1.2555	1.1501	1.3704	0.0561	2775.10	1.3671	1.1386	1.6415	0.1276	845.16
		scale	20.1901	18.5692	21.9525	0.8621		22.8177	18.5265	28.1028	2.4254	
	Gamma	shape	1.4723	1.2891	1.6815	0.0998	2768.85	1.5097	1.1925	1.9113	0.1817	844.28
		rate	0.0776	0.0651	0.0926	0.0070		0.0672	0.0443	0.1020	0.0143	
	Exp	rate	0.0490	0.0441	0.0545	0.0026	2796.57	0.0323	0.0264	0.0394	0.0033	853.10
	Log-logistic	shape	1.6930	1.5514	1.8475	0.0754	2759.36	1.5145	1.2655	1.8125	0.1388	843.38
		scale	13.8408	12.5886	15.2175	0.6696		18.5865	15.1847	22.7504	1.9170	
	Log-normal	meanlog	2.6216	2.5227	2.7206	0.0505	2766.82	3.0426	2.8013	3.2839	0.1231	844.11
		sdlog	1.0369	0.9593	1.1208	0.0412		1.2691	1.0807	1.4903	0.1041	
	Gompertz	shape	0.0161	0.0038	0.0284	0.0063	2792.22	0.0600	0.0043	0.1156	0.0284	850.77
		rate	0.0410	0.0343	0.0490	0.0037		0.0232	0.0158	0.0341	0.0045	
	Royston/Parmar spline model (0 knot)	gamma0	-3.7728	-4.1195	-3.4261	0.1769	2775.10	-4.2757	-4.8886	-3.6628	0.3127	845.16
		gamma1	1.2554	1.1454	1.3654	0.0561		1.3671	1.1171	1.6172	0.1276	
	Royston/Parmar spline model (1 knot)	gamma0	-4.4832	-5.0274	-3.9391	0.2776	2760.44	-4.5727	-5.3891	-3.7563	0.4165	845.50
		gamma1	2.4603	1.8178	3.1029	0.3278		1.9805	0.9432	3.0178	0.5292	
		gamma2	0.0759	0.0372	0.1147	0.0198		0.0887	-0.0529	0.2302	0.0722	
	Royston/Parmar spline model (2 knot)	gamma0	-4.4486	-4.9899	-3.9074	0.2762	2762.29	-4.6034	-5.4045	-3.8022	0.4088	846.60
		gamma1	2.2368	1.2381	3.2356	0.5096		1.5602	0.2825	2.8379	0.6519	
		gamma2	-0.0348	-0.2634	0.1937	0.1166		-0.3031	-1.0000	0.3937	0.3555	
		gamma3	0.1342	-0.1606	0.4289	0.1504		0.5613	-0.4766	1.5992	0.5296	
	Mixture cure model (Weibull)	theta	0.1247	0.0717	0.2081	NA	2769.66	0.5621	0.3979	0.7138	NA	843.64
		shape	1.3998	1.2584	1.5571	0.0760		1.6960	1.3323	2.1589	0.2088	
		scale	16.2332	14.2225	18.5282	1.0952		9.6355	6.5162	14.2480	1.9230	
	Mixture cure model (Gamma)	theta	0.1077	0.0552	0.1998	NA	2764.92	0.4802	0.2458	0.7237	NA	844.14
		shape	1.6888	1.4308	1.9933	0.1428		1.9064	1.3181	2.7572	0.3589	
		rate	0.1092	0.0827	0.1442	0.0155		0.1736	0.0712	0.4231	0.0789	
	Mixture cure model (Exp)	theta	0.0001	0.0000	1.0000	NA	2798.57	0.0007	0.0000	1.0000	NA	855.11
		rate	0.0490	0.0441	0.0545	0.0026		0.0323	0.0264	0.0396	0.0034	
	Mixture cure model (Log-logistic)	theta	0.0031	0.0000	1.0000	NA	2761.38	0.3783	0.1186	0.7335	NA	844.08
		shape	1.6978	1.5388	1.8733	0.0852		1.7579	1.3163	2.3477	0.2595	
		scale	13.7799	12.2441	15.5083	0.8308		11.0251	5.8470	20.7889	3.5677	
	Mixture cure model (Log-normal)	theta	0.0004	0.0000	1.0000	NA	2768.84	0.0024	0.0000	1.0000	NA	846.12
		meanlog	2.6210	2.5214	2.7207	0.0508		3.0395	2.7646	3.3144	0.1403	
		sdlog	1.0367	0.9588	1.1208	0.0413		1.2680	1.0762	1.4942	0.1062	

© 2021 Su D et al. *JAMA Network Open*.

	Mixture cure model (Gompertz)	theta	0.1331	0.0772	0.2196	NA	2789.79	0.6198	0.5355	0.6975	NA	845.84
		shape	0.0406	0.0202	0.0611	0.0104		0.2128	0.1298	0.2958	0.0423	
		rate	0.0423	0.0351	0.0511	0.0041		0.0414	0.0264	0.0651	0.0096	
	Non-mixture cure model (Weibull)	theta	0.0974	0.0409	0.2143	NA	2765.50	0.5432	0.3431	0.7303	NA	843.77
		shape	1.5157	1.3484	1.7036	0.0904		1.7265	1.3431	2.2194	0.2212	
		scale	28.0300	19.4237	40.4495	5.2454		11.3519	6.3986	20.1397	3.3205	
	Non-mixture cure model (Gamma)	theta	0.0750	0.0266	0.1942	NA	2763.15	0.4410	0.1735	0.7478	NA	844.20
		shape	1.6993	1.4339	2.0139	0.1473		1.8742	1.2862	2.7309	0.3600	
		rate	0.0562	0.0320	0.0988	0.0162		0.1285	0.0379	0.4359	0.0801	
	Non-mixture cure model (Exp)	theta	0.0000	0.0000	1.0000	NA	2799.37	0.0000	0.0000	1.0000	NA	855.36
		rate	0.0010	0.0002	0.0048	0.0008		0.0018	0.0000	3.7178	0.0070	
	Non-mixture cure model (Log-logistic)	theta	0.0443	0.0114	0.1576	NA	2763.56	0.3866	0.1368	0.7148	NA	844.05
		shape	1.6042	1.3963	1.8429	0.1136		1.7501	1.3087	2.3404	0.2595	
		scale	30.3637	19.0768	48.3285	7.2003		14.1656	5.9057	33.9779	6.3233	
	Non-mixture cure model (Log-normal)	theta	0.0000	0.0000	0.9900	NA	2762.82	0.0010	0.0000	1.0000	NA	845.24
		meanlog	5.0858	3.1088	7.0628	1.0087		5.2067	-0.9780	11.3913	3.1555	
		sdlog	1.6321	1.2067	2.2072	0.2514		1.7375	0.8117	3.7193	0.6747	
	Non-mixture cure model (Gompertz)	theta	0.1338	0.0776	0.2209	NA	2787.93	0.6193	0.5345	0.6974	NA	845.67
		shape	0.0630	0.0404	0.0857	0.0116		0.2308	0.1464	0.3152	0.0431	
		rate	0.0174	0.0133	0.0228	0.0024		0.0321	0.0199	0.0516	0.0078	
Atezolizumab plus bevacizumab	Weibull	shape	1.2138	1.1131	1.3237	0.0537	2733.08	1.2728	1.0270	1.5773	0.1393	512.57
		scale	18.9127	17.3445	20.6227	0.8352		16.2507	12.9786	20.3479	1.8642	
	Gamma	shape	1.4438	1.2641	1.6491	0.0979	2723.80	1.4076	1.0556	1.8770	0.2067	511.92
		rate	0.0813	0.0682	0.0971	0.0073		0.0897	0.0561	0.1435	0.0215	
	Exp	rate	0.0525	0.0473	0.0583	0.0028	2748.69	0.0525	0.0412	0.0670	0.0065	514.99
	Log-logistic	shape	1.6865	1.5463	1.8394	0.0747	2699.59	1.4717	1.1937	1.8146	0.1573	511.22
		scale	12.5778	11.4312	13.8394	0.6134		12.3488	9.7877	15.5801	1.4645	
	Log-normal	meanlog	2.5401	2.4439	2.6363	0.0491	2693.32	2.5567	2.2918	2.8215	0.1351	510.89
		sdlog	1.0093	0.9335	1.0912	0.0402		1.2314	1.0197	1.4870	0.1185	
	Gompertz	shape	0.0059	-0.0069	0.0187	0.0065	2749.88	0.0465	-0.0222	0.1152	0.0351	515.28
		rate	0.0494	0.0416	0.0587	0.0043		0.0415	0.0266	0.0645	0.0094	
	Royston/Parmar spline model (0 knot)	gamma0	-3.5684	-3.8955	-3.2413	0.1669	2733.08	-3.5487	-4.2056	-2.8917	0.3352	512.57
		gamma1	1.2138	1.1086	1.3190	0.0537		1.2728	0.9997	1.5458	0.1393	
	Royston/Parmar spline model (1 knot)	gamma0	-5.1176	-5.7859	-4.4492	0.3410	2692.84	-3.8625	-4.6972	-3.0277	0.4259	512.18
		gamma1	2.4528	2.0122	2.8933	0.2248		1.9988	0.9548	3.0428	0.5327	
		gamma2	0.1515	0.1026	0.2004	0.0250		0.0989	-0.0326	0.2304	0.0671	
	Royston/Parmar spline model (2 knot)	gamma0	-5.2768	-6.1235	-4.4301	0.4320	2694.52	-3.8714	-4.7021	-3.0407	0.4238	514.05
		gamma1	2.6445	1.9094	3.3797	0.3751		1.8750	0.4100	3.3401	0.7475	

© 2021 Su D et al. *JAMA Network Open*.

		gamma2	0.1633	-0.1172	0.4437	0.1431		-0.0644	-0.7659	0.6371	0.3579	
		gamma3	-0.0131	-0.3252	0.2989	0.1592		0.1943	-0.6946	1.0833	0.4535	
	Mixture cure model (Weibull)	theta	0.1717	0.1276	0.2271	NA	2710.72	0.2874	0.0460	0.7713	NA	514.01
		shape	1.4675	1.3310	1.6180	0.0731		1.3891	1.0415	1.8527	0.2041	
		scale	13.5815	12.2713	15.0316	0.7030		10.8445	5.1269	22.9387	4.1452	
	Mixture cure model (Gamma)	theta	0.1631	0.1182	0.2207	NA	2702.06	0.2670	0.0389	0.7660	NA	513.35
		shape	1.9013	1.6207	2.2304	0.1549		1.5995	1.0468	2.4439	0.3460	
		rate	0.1511	0.1197	0.1906	0.0179		0.1505	0.0489	0.4631	0.0863	
	Mixture cure model (Exp)	theta	0.0067	0.0000	1.0000	NA	2750.68	0.0006	0.0000	1.0000	NA	516.99
		rate	0.0532	0.0425	0.0667	0.0061		0.0526	0.0411	0.0672	0.0066	
	Mixture cure model (Log-logistic)	theta	0.0872	0.0374	0.1903	NA	2697.27	0.1425	0.0014	0.9520	NA	513.07
		shape	1.8660	1.6520	2.1079	0.1160		1.5618	1.0951	2.2274	0.2829	
		scale	10.9991	9.5937	12.6103	0.7671		10.1632	4.2010	24.5872	4.5810	
	Mixture cure model (Log-normal)	theta	0.0671	0.0170	0.2298	NA	2693.64	0.0008	0.0000	1.0000	NA	512.89
		meanlog	2.4289	2.2573	2.6005	0.0876		2.5555	2.2813	2.8298	0.1399	
		sdlog	0.9413	0.8317	1.0653	0.0594		1.2310	1.0182	1.4882	0.1192	
	Mixture cure model (Gompertz)	theta	0.1720	0.1271	0.2286	NA	2739.58	0.1605	0.0000	0.9996	NA	517.26
		shape	0.0475	0.0287	0.0664	0.0096		0.0623	-0.1132	0.2379	0.0896	
		rate	0.0501	0.0416	0.0602	0.0047		0.0485	0.0121	0.1953	0.0345	
	Non-mixture cure model (Weibull)	theta	0.1575	0.1088	0.2227	NA	2703.99	0.2624	0.0280	0.8146	NA	513.58
		shape	1.6171	1.4592	1.7922	0.0848		1.4559	1.0805	1.9615	0.2215	
		scale	19.9994	16.4474	24.3186	1.9953		15.5044	3.9797	60.4035	10.7577	
	Non-mixture cure model (Gamma)	theta	0.1424	0.0930	0.2119	NA	2698.65	0.2396	0.0258	0.7894	NA	513.15
		shape	1.9556	1.6662	2.2953	0.1598		1.6193	1.0558	2.4834	0.3533	
		rate	0.0984	0.0682	0.1418	0.0184		0.1001	0.0177	0.5676	0.0886	
	Non-mixture cure model (Exp)	theta	0.0000	0.0000	1.0000	NA	2751.13	0.0000	0.0000	1.0000	NA	517.15
		rate	0.0014	0.0002	0.0092	0.0014		0.0022	0.0001	0.0664	0.0038	
	Non-mixture cure model (Log-logistic)	theta	0.1048	0.0592	0.1788	NA	2699.35	0.1850	0.0126	0.8013	NA	513.22
		shape	1.7875	1.5747	2.0291	0.1156		1.5504	1.0840	2.2176	0.2831	
		scale	19.2839	14.7142	25.2727	2.6610		15.9739	3.6614	69.6900	12.0059	
	Non-mixture cure model (Log-normal)	theta	0.0444	0.0102	0.1732	NA	2693.41	0.0023	0.0000	1.0000	NA	512.62
		meanlog	3.3939	2.8031	3.9847	0.3014		4.5780	-0.9138	10.0697	2.8019	
		sdlog	1.1619	0.9635	1.4011	0.1110		1.6924	0.7839	3.6540	0.6646	
	Non-mixture cure model (Gompertz)	theta	0.1701	0.1260	0.2258	NA	2737.36	0.2190	0.0019	0.9763	NA	517.17
		shape	0.0728	0.0530	0.0926	0.0101		0.0940	-0.0748	0.2628	0.0861	
		rate	0.0223	0.0178	0.0280	0.0026		0.0260	0.0023	0.2923	0.0321	

© 2021 Su D et al. *JAMA Network Open*.

eTable 3. Probability and Costs Related to Adverse Events (Grade ≥3)

Parameters	Expected value	Range	Distribution	Reference
Probabilities in sorafenib arm				(5)
Diarrhea	0.05	0.42 - 0.79	Beta: α= 0.3, β= 5.2	
Palmar–plantar erythrodysesthesia syndrome	0.08	0.06 - 0.10	Beta: α= 14.7, β= 162.1	
Fatigue	0.03	0.02 - 0.04	Beta: α= 15.5, β= 468.5	
Nausea	0.01	0.00 - 0.01	Beta: α= 15.9, β= 2634.8	
Hypertension	0.12	0.09 - 0.15	Beta: α= 14, β= 101.1	
Thrombocytopenia	0.01	0.01 - 0.02	Beta: α= 15.8, β= 1199	
Hepatotoxicity	0.06	0.05 - 0.08	Beta: α= 15, β= 219	
Proteinuria	0.01	0.00 - 0.01	Beta: α= 15.9, β= 2634.8	
Probabilities in atezolizumab plus bevacizumab arm				(5)
Diarrhea	0.02	0.01 - 0.02	Beta: α= 15.7, β= 857.2	
Palmar–plantar erythrodysesthesia syndrome	0.00	0.00 - 0.00	Beta: α= 0, β= 0	
Fatigue	0.02	0.02 - 0.03	Beta: α= 15.6, β= 635.1	
Nausea	0.00	0.00 - 0.00	Beta: α= 16, β= 5301.4	
Hypertension	0.15	0.11 - 0.19	Beta: α= 13.6, β= 75.7	
Thrombocytopenia	0.03	0.02 - 0.04	Beta: α= 15.5, β= 453.4	
Hepatotoxicity	0.11	0.08 - 0.13	Beta: α= 14.3, β= 120.6	
Proteinuria	0.03	0.02 - 0.04	Beta: α= 15.5, β= 501.8	
Costs per event				
Diarrhea	3,802	2,851 - 4,752	Gamma: α= 15206, λ= 0.25	(13)
Palmar–plantar erythrodysesthesia syndrome	987	741 - 1,234	Gamma: α= 3949, λ= 0.25	(13)
Fatigue	249	187 - 311	Gamma: α= 996, λ= 0.25	(13)
Nausea	2,638	1,978 - 3,297	Gamma: α= 10551, λ= 0.25	(13)
Hypertension	1,701	1,276 - 2,127	Gamma: α= 6805, λ= 0.25	(13)
Thrombocytopenia	4,094	1,750 - 9,579	Gamma: α= 8390, λ= 0.488	(12)
Hepatotoxicity	2,773	-1,584 - 7,119	Gamma: α= 3461, λ= 0.801	(15)
Proteinuria	1,728	97 - 3,565	Gamma: α= 3376, λ= 0.512	(15)

© 2021 Su D et al. *JAMA Network Open*.
